# Supplementary material for: Better late than never: Optimising the proteomic analysis of field-collected octopus
Source: PLoS One. 2023 Jul 12;18(7):e0288084. doi: 10.1371/journal.pone.0288084 (PMC10337964; doi:10.1371/journal.pone.0288084)
Supplement: S1 Table — (PDF) [file pone.0288084.s001.pdf]

Title

Better late than never: optimising the proteomic analysis of field-collected octopus

Authors

Qiaz Q.H. Hua, Clifford Young, Tara L. Pukala, Peter Hoffmann, Jasmin C. Martino, Bronwyn M. Gillanders, Zoe A. Doubleday

S1 Table. Recipe for homemade RNA*later* and RIPA buffer.

Chemical Solution	Ingredient List & Quantity
**RNA*later***	• 935 ml of nuclease-free and protease-free water (Acros Organics)<br>• 1.4 kg of ammonium sulfate dihydrate (Univar Solutions)<br>• 40 ml of 0.5 M Ultrapure EDTA, pH 8 (ThermoFisher)<br>• 25 ml of 1 M sodium citrate (Chem-Supply)<br><br>Combine the above ingredients and stir on low heat until the ammonium sulfate has dissolved. Allow it to cool. Adjust the pH of the solution to 5.2 by using 1 M sulfuric acid (Sigma Aldrich). Store at room temperature or in the fridge.
**RIPA Buffer**	• 150 mM NaCl (Merck)<br>• 1% Igepal (Sigma Aldrich)<br>• 0.5% sodium deoxycholate (Sigma Aldrich)<br>• 0.2% SDS (GE Healthcare)<br>• 50 mM Tris pH 8 (Astral Scientific)<br>• Protease inhibitor cocktail 1/100 (Sigma Aldrich)<br>• 20 mM dithiothreitol (Roche)<br><br>Combine all above ingredients (except for protease inhibitor) and mix well. Store at -20°C. Just before using the buffer, add the protease inhibitor to the solution.
