# Supplementary material for: Better late than never: Optimising the proteomic analysis of field-collected octopus
Source: PLoS One. 2023 Jul 12;18(7):e0288084. doi: 10.1371/journal.pone.0288084 (PMC10337964; doi:10.1371/journal.pone.0288084)
Supplement: S2 Table — (PDF) [file pone.0288084.s002.pdf]

Title

Better late than never: optimising the proteomic analysis of field-collected octopus

Authors

Qiaz Q.H. Hua, Clifford Young, Tara L. Pukala, Peter Hoffmann, Jasmin C. Martino, Bronwyn M. Gillanders, Zoe A. Doubleday

S2 Table. List of muscular proteins that were significantly higher in abundance in samples homogenised using metal beads compared to homogenisation with LN2.

UniProt Accession No.	Protein Name	Abundance Ratio: Beads/LN_2	Adjusted p-value
A0A6P7S485	Actin-related protein 3	3.15	0.018
A0A0L8HI19	Actin-related protein 2/3 complex subunit	2.39	0.039
A0A0L8FI18	Actin-related protein 2/3 complex subunit 4	2.77	0.031
A0A6P7SD63	Actin-like protein 6B	2.70	0.044
A0A6P7TGU1	Actin-interacting protein 1 isoform	2.39	0.028
A4D0I0	Beta actin	2.99	0.039
A0A6P7SR66	Myophilin	2.45	0.049
Q6E216	Tropomyosin-like protein	3.28	0.014
A0A7E6ET58	Tropomyosin Tod p 1.0102 isoform X11	5.77	0.039
