# Supplementary material for: Better late than never: Optimising the proteomic analysis of field-collected octopus
Source: PLoS One. 2023 Jul 12;18(7):e0288084. doi: 10.1371/journal.pone.0288084 (PMC10337964; doi:10.1371/journal.pone.0288084)
Supplement: S1 Raw images — (PDF) [file pone.0288084.s007.pdf]

Title

Better late than never: optimising the proteomic analysis of field-collected octopus

Authors

Qiaz Q.H. Hua, Clifford Young, Tara L. Pukala, Peter Hoffmann, Jasmin C. Martino, Bronwyn M. Gillanders, Zoe A. Doubleday

Supplementary Material

Method of Image Capture:

Samples were loaded into a pre-cast NuPAGETM 4 to 12% Bis-Tris polyacrylamide gel (Thermo Fisher Scientific). Gels were run at 180 V for 60 min and fixed briefly before staining overnight with Coomassie Brilliant Blue G-250 (Sigma Aldrich, Burlington, USA). Upon de-staining with Milli-Q water, the gels were imaged on a GelDoc Imaging System (Bio-Rad, Hercules, USA).

Legend:

WoR0: Sample without RNAlater (0h)

WoR3: Sample without RNAlater at 4C (3h)

WoRT3: Sample without RNAlater at room temp (3h)

WoR6: Sample without RNAlater (6h)

WoRT6: Sample without RNAlater at room temp (6h)

HR0: Sample with RNAlater (0h)

HR3: Samples with RNAlater at 4C (3h)

HR6: Sample with RNAlater at 4C (6h)

CR0: Sample with commercial RNAlater (0h)

CR3: Samples with RNAlater at 4C (3h)

CR6: Sample with commercial RNAlater at 4C (6h)

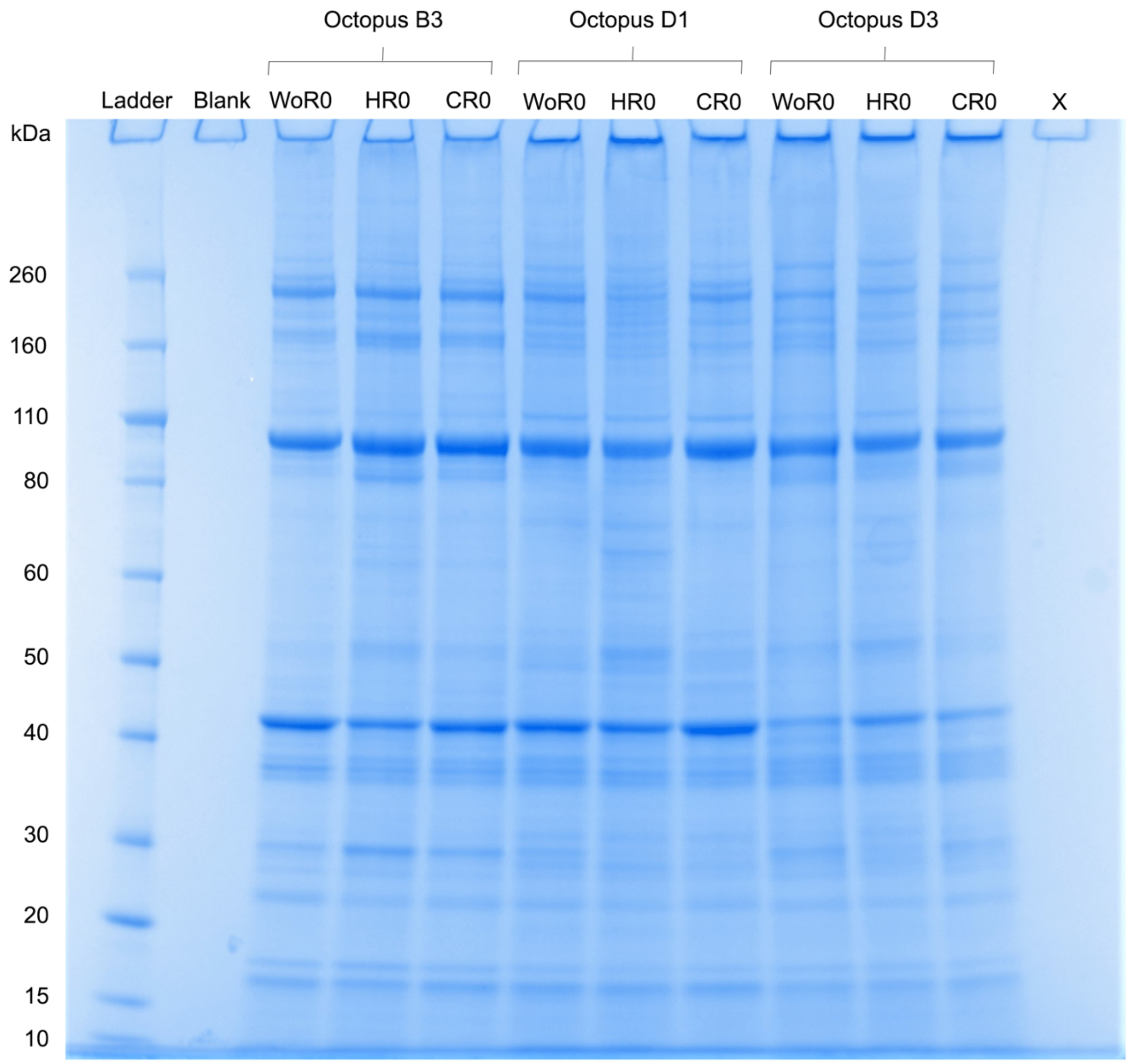

Fig S1a. Original gel image corresponding to Fig 2a in the main text.

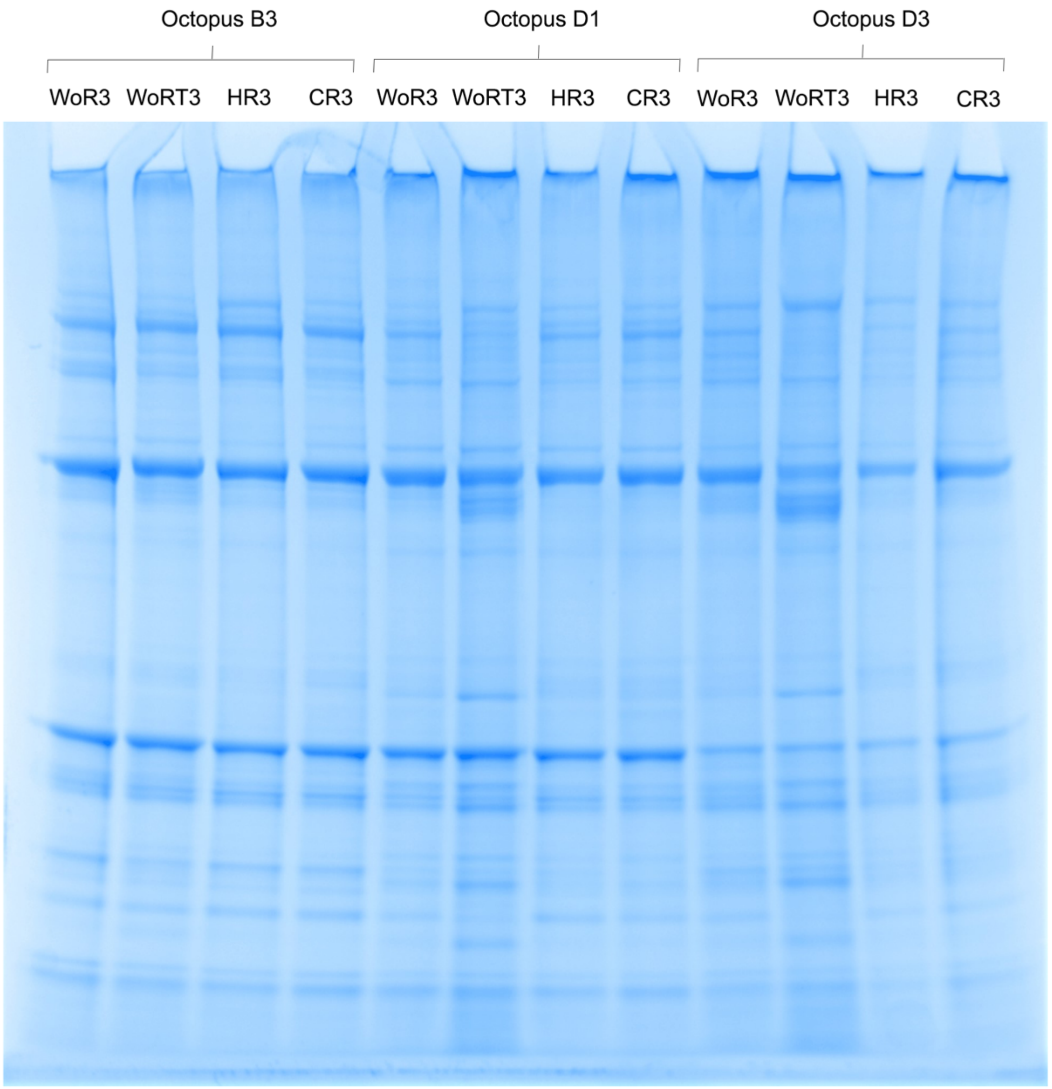

Fig S1b. Original gel image corresponding to Fig 2b in the main text.

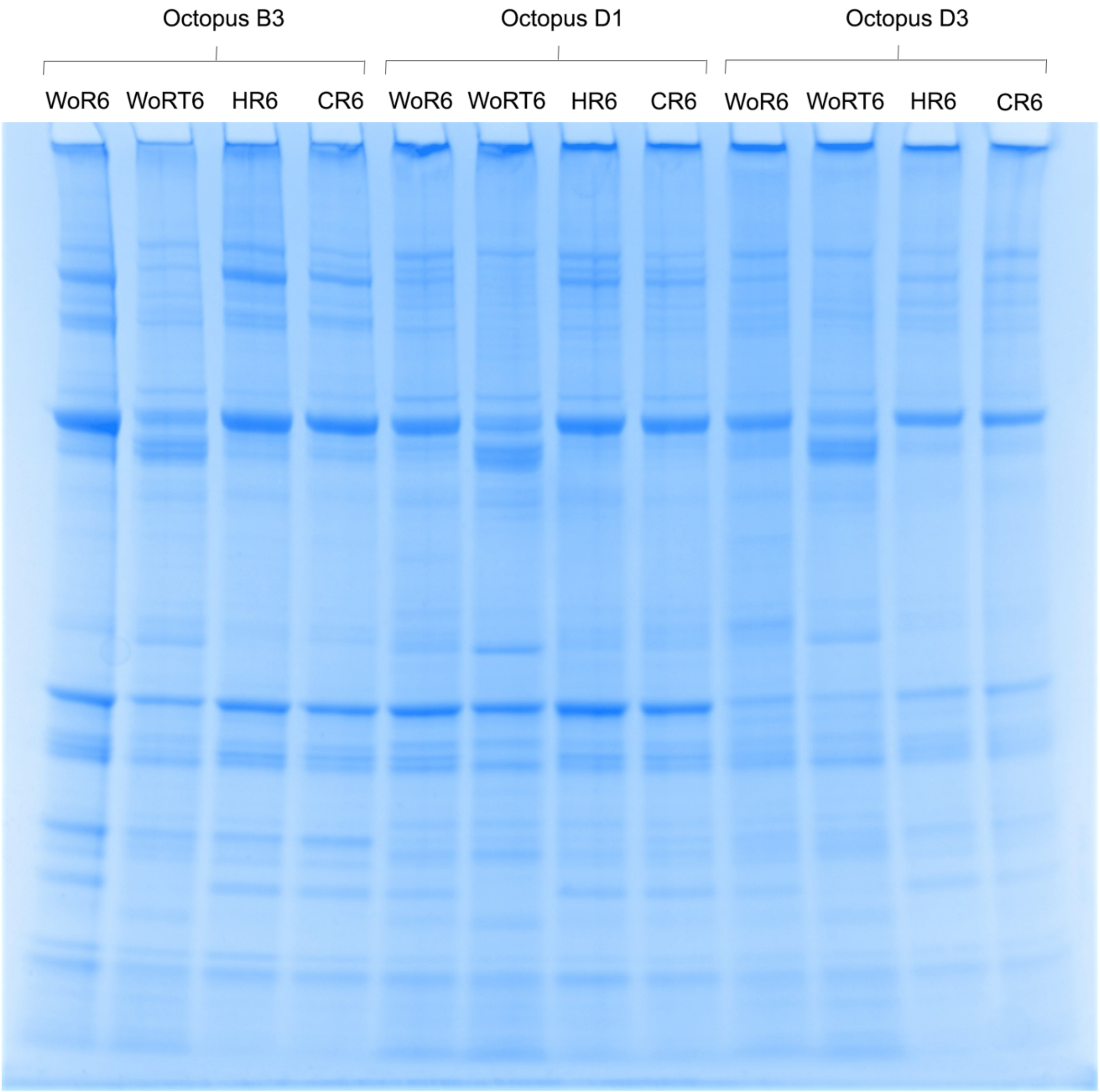

Fig S1c. Original gel image corresponding to Fig 2c in the main text.
