# Supplementary material for: Better late than never: Optimising the proteomic analysis of field-collected octopus
Source: PLoS One. 2023 Jul 12;18(7):e0288084. doi: 10.1371/journal.pone.0288084 (PMC10337964; doi:10.1371/journal.pone.0288084)
Supplement: Attachment — Submitted filename: PlosOne_ResponseToReviewers.docx [file pone.0288084.s008.docx]

**Response to Reviewers**

We would like to thank the reviewers for their valuable feedback and have provided our responses below in blue. All new Line numbers are in reference to the Revised Manuscript with Track Changes.

Reviewer #1: Authors reported a study on the optimization of sample collection and processing for proteomic analysis of field-collected octopus. The study design is straight-forward and is relevant to answer the research question. However, there are a number of issues which compromise the quality of the paper and authors should attempt to address these to satisfaction before the manuscript can be considered for publication.

1. Title: it should be field-collected octopus -- as appeared in the title stated in other parts of the submission (supplementary files). Authors only performed the work on octopus, not all representative organisms.
Thank you for the suggestion. We have amended the title to ‘octopus’ instead of ‘organisms’.

2. I understand that authors wanted to study the "proteomics" of "octopus" using the octopus tissues which were stored and processed differently (hence the optimizing methods). However authors need to be clear in the beginning and state in the introduction what "proteomes" are they looking for, and subsequently the justification of the materials and techniques applied.

Thank you for the comment. We have clarified this in the introduction by making the following changes (new Lines 137 to 143):

“In this study, we optimised proteomic methods for both sample preservation and preparation using remote field-caught octopus. To fulfil our aims of method optimisation, we examined the arm proteome as octopus arms can be rapidly sampled both in the laboratory and field compared to other tissues such as organs. Given that existing protocols typically use soft tissues, muscular tissues like octopus arms require further optimisation to ensure efficient protein extraction.”

3. Other suggestions for introduction:
Line 77: what is traceability of seafood - this seems a bit out of place in the context of this statement.
Also, in the context of animal studies, proteomics has been widely applied in snakes to unravel the venom complexity and diversity including intra- and inter-species variation, which carries ecological, evolutionary and medical significance. This part of information should be included. Suggest references (some examples of reviews on snake venomics):
- <https://www.frontiersin.org/articles/10.3389/fphar.2021.768015/full>
- <https://www.mdpi.com/2072-6651/14/4/247>
- <https://www.ncbi.nlm.nih.gov/pmc/articles/PMC5408369/>

Thank you for the comments. The study of seafood traceability examines where seafood originates, and proteomics tools have been applied in this field to ensure authenticity of seafood. We have changed the term to “seafood provenance” for clarity (new Line 89). We have also included two of the suggested references for venomics (new Lines 89 to 90; References #6 and #8).

Line 80-81: ... proteomics-based in ecology - please be specific regarding which aspect of ecology studies authors feel that proteomics is lacking?
Thank you for the suggestion. We have added more details as follows (new Lines 92 to 96):

“Despite its clear advantages and broad applications, there is a relative lack of ecological studies that use proteomics to understand human impacts and stressors, such as global warming and ocean acidification.”

Line 97-99: storage solutions - what about undenatured ethanol? This is a very commonly and easy-to-use method for tissue sampling in the wild. Authors should include this in the introduction and provide justification.
Thank you for the suggestion. While we agree that undenatured ethanol is common and easy to use for tissue sampling in the wild, we’ve focussed on non-hazardous liquids to further reduce risks and simplify logistics. We have made the following changes (new Lines 111 to 113):

“The use of LN2 and ethanol also poses a safety concern in the field especially for long-distance travel or in the confined spaces of a vehicle.”

4. Methods:
Line 136: How long were the octopus were kept and raised in aquaria?
The adult octopuses were kept in aquaria for three months until spawning and hatching of embryos. We have included this in the text (new Lines 159 to 161) as follows:

“Three female *Octopus berrima* (biological replicates) were collected directly from a commercial fishery in South Australia and raised in aquaria for three months until spawning and hatching of embryos.”

Line 140-141: .......arm sections were dissected and immediately stored at -20 C... was this in the presence of RNAlatere? What is the ratio of RNAlater to the tissue?

The arm sections were initially stored without RNAlater at -20C, so that comparisons can be made using arm sections with or without RNAlater (i.e. control). After which, 5X volume of RNAlater was added to about 10 mg of tissues (new Lines 181 to 182, and 185).

Line 142... maintained at 4 C. with those at room temperature without RNAlater... the description is unclear. What is the volume, duration, amount of tissue etc.... these technical information needs to be included and described clearly.
Thank you for the suggestion. 10 mg of tissue per sample were used and 5X volume of RNAlater was added to each tissue. The duration depends on the condition i.e. 3 hours, 6 hours, or a few seconds for the “immediate” condition.

We have made the following changes (new Lines 181 to 183):

“For samples with RNAlater, 5X volume of RNAlater was added to each arm section for the durations stated above.”

Section: Proteomics
- How did authors define "protein abundances"? Do authors mean relative fold-change in "protein expression"? How was this measured, is it by the number of proteins identified per sample, or by the amount of protein (in terms of relative weight) per sample? Please provide the quantitative determination of the protein abundance for each proteome.
According to the software developed by Thermo Fisher, peptide group abundances are based on the intensity of their precursors, and protein abundances are calculated as the simple summation of their associated peptide group abundances. Protein abundance is not equivalent to protein expression, as the latter does not take into account protein turnover. Since we are only measuring and comparing protein abundances, we have therefore removed all instances of “protein expression” in the manuscript to avoid any confusion. The same amount of peptide samples (i.e. 1 µg per sample) was used for mass spectrometry to ensure fair comparisons. After which, at the bioinformatic stage, relative protein abundances were compared across the different conditions. The number of proteins identified per sample was also included in the comparisons.

We added details in the Methods under “Data analysis” as follows (new Lines 339 to 342):

“Peptide group abundances were based on the intensity of their precursors, while protein abundances were calculated as the summation of their associated peptide group abundances.”

As a common practice in sequencing and proteomics/genomics study, the raw data of MS should be deposited and archived in a repository for public access. If this is available, please provide the repository accordingly.
Thank you for the advice. We have deposited all data in the PRIDE repository but we are currently awaiting an accession number due to technical difficulties. We will provide the number to the journal as soon as we receive it.

Results and Discussion:
Provide the statistical significance of all parameters used in comparison, in tables and figures.

Thank you for the comment. Specific p-values can be found in the Supporting Information for each protein and we have clarified the supplementary captions for easier location of the p-values. Overall, the results that have been shown have already been filtered in the software such that only comparisons with an adjusted p-value of < 0.05 are shown i.e. only the skin comparison showed significant differences.

Suggest to include limitations of the work.

We have originally discussed the limitations of our work and these can be found in the Discussion at new Lines 492 to 512 and 541 to 561.

Reviewer #2: Dear authors, thank you for the opportunity to review such a clearly and convincingly written paper. Unfortunately, I am an ecologist and am far from qualified to judge whether the chemistry or bioinformatics were appropriate for the task. Indeed, probably half of the paper, from the methods through the results could have been completely made up and I wouldnt be able to tell. However, given the clarity of the writing and knowing that Zoe Doubleday and Bronwyn Gillanders were part of the research and the paper, I fully trust that this was the not case.

We thank the reviewer for their trust in our work. The proteomics-based methods were developed from established protocols alongside our co-authors Young, Pukala and Hoffmann, who are experts in the field of proteomics (see example references below). We have deposited all our data (awaiting accession number) and will make it publicly available upon acceptance, such that other researchers can also use our data in future assessments and validate our bioinformatic findings.

- Williams, D.M. and Pukala, T.L. (2013), Novel insights into protein misfolding diseases revealed by ion mobility-mass spectrometry. Mass Spectrom. Rev., 32: 169-187. <https://doi.org/10.1002/mas.21358>
- Tara L. Pukala; Mass spectrometric insights into protein aggregation. Essays Biochem 29 March 2023; 67 (2): 243–253. doi: <https://doi.org/10.1042/EBC20220103>
- Sylvestersen, K.B., Young, C., Nielsen, M.L. (2013), Advances in characterizing ubiquitylation sites by mass spectrometry. Current Opinion in Chemical Biology., 17(1): 49–58. doi: <https://doi.org/10.1016/j.cbpa.2012.12.009>
- Donnellan L, Young C, Simpson BS, Dhillon VS, Costabile M, Hoffmann P, Fenech M, Deo P. Methylglyoxal Impairs Sister Chromatid Separation in Lymphocytes. International Journal of Molecular Sciences. 2022; 23(8):4139. https://doi.org/10.3390/ijms23084139

I had a couple of questions/comments:
I might have missed it (or misunderstood), in which case it could be made clearer, but I was a little uncertain as to what the RNAlater preserved samples were compared against. This confusion arose somewhere around the first sentence of the methods. I understood that they were compared against samples that were not treated with RNAlater, but wouldnt it have made most sense to also understand how they performed against the current lab best-practice for handling of samples for proteomic analyses, presumably freezing immediately at -80C (or being treated with some other less-transportable preservative)? Its not a deal-breaker but might be worth clarifying for a non-proteomics expert if/where you think it would be appropriate in the paper.

Thank you for the suggestions. The samples in RNAlater were compared against the current lab best-practice i.e. -20°C preservation. These best-practice samples are equivalent to the “immediate” samples without RNAlater that served as the control in the gel analysis.

We have edited the methods for the Optimisation of Test Samples with RNA*later* (first paragraph of Methods) for clarity, as follows (new Lines 159 to 183):

“Three female Octopus berrima (biological replicates) were collected directly from a commercial fishery in South Australia and raised in aquaria for three months until spawning and hatching of embryos. They were then euthanised in the lab in accordance with the Australian code for the care and use of animals for scientific purposes [21] by exposure to 1.5% magnesium chloride for 10 min, followed by exposure to 3.5% magnesium chloride for 30 min. Arm sections were dissected and immediately stored at -20 °C, which is a recommended temperature for storage of proteomics samples and these samples were hence used as the control in this study. To determine if RNAlater incubation helped maintain protein integrity, we compared samples from three timepoints: immediately, after 3 h and 6 h in the presence or absence of RNAlater, during which samples were maintained at 4 °C. These timepoints were based on the time necessary to complete the fieldwork. We then compared the effect of homemade (S1 Table) and commercial RNAlater (Thermo Fisher Scientific, Waltham, USA) on protein quality and quantity. We additionally compared these samples maintained at 4 °C without RNAlater with those incubated at room temperature without RNAlater to ascertain if cold storage was sufficient for maintaining protein quality. For samples with RNAlater, 5X volume of RNAlater was added to each arm section for the durations stated above.”

Very minor detail: the wording of the sentence L86-88 is a little strange and sounds like the sampling sites are inside a moving car. It would be worth adjusting the wording there slightly.

Thank you for the suggestion. We have clarified the writing by making the following changes (new Line 102 to 104):

“For instance, field sampling sites may be remote and sampling may take place within awkward, confined spaces, such as a moving boat, where only ice may be available.”

Other than that, well done!, and I hope that the second reviewer had some knowledge in proteomics... or at least chemistry... or bioinformatics... to be able to give you feedback on those sections of the manuscript.
All the best...
